# Enhanced Stability and Bioavailability of Defatted Cricket Protein Hydrolysates Encapsulated in Alginate-Coated Liposomes

**DOI:** 10.3390/foods15081345

**Published:** 2026-04-13

**Authors:** Lalita Chotphruethipong, Soottawat Benjakul, Rotimi E. Aluko, Theeraphol Senphan, Pilaiwanwadee Hutamekalin, Sirima Sinthusamran

**Affiliations:** 1 Department of Food Science, Faculty of Science, Burapha University, Mueang Chonburi, Chonburi 20131, Thailand; 2 International Center of Excellence in Seafood Science and Innovation, Faculty of Agro-Industry, Prince of Songkla University, Hat Yai, Songkhla 90110, Thailand; soottawat.b@psu.ac.th; 3 Department of Food and Human Nutritional Sciences, University of Manitoba, Winnipeg, MB R3T 2N2, Canada; rotimi.aluko@umanitoba.ca; 4 Program in Food Science and Technology, Faculty of Engineering and Agro-Industry, Maejo University, Sansai, Chiangmai 50290, Thailand; theeraphol_s@mju.ac.th; 5 Division of Health and Applied Sciences, Faculty of Science, Prince of Songkla University, Hat Yai, Songkhla 90110, Thailand; pilaiwanwadee.h@psu.ac.th; 6 Department of Agricultural Education, School of Industrial Education and Technology, King Mongkut’s Institute of Technology Ladkrabang, Bangkok 10520, Thailand; sirima.ta@kmitl.ac.th

**Keywords:** cricket, protein hydrolysate, liposome, alginate, coating, stability, bioavailability

## Abstract

The practical application of protein hydrolysates as functional food ingredients is frequently obstructed by their inherent structural instability. To circumvent this limitation, liposomal encapsulation has emerged as a sophisticated strategy to bolster the bioactivity and integrity of cricket-derived proteins. In this study, varying concentrations (1–4% *w*/*v*) of defatted cricket protein hydrolysate (DCPH) were integrated into vesicles composed of soy lecithin and cholesterol. The highest encapsulation efficiency (EE) was observed at a 2% DCPH loading capacity, yielding a significant result of 88.18% (*p* < 0.05). Subsequent coating with sodium alginate (SA) at 0.1–0.3% (*w*/*v*) resulted in an increase in particle size and a more pronounced negative surface charge. When maintained at 4 °C over a 24-day duration, the SA-coated liposome (SA-L-2%DCPH) exhibited superior stability compared to its uncoated (L-2%DCPH) counterpart. Also, the digest derived from the SA-L-2%DCPH exhibited significantly enhanced transepithelial permeability across the Caco-2 cell monolayer, indicated by the higher protein content and ABTS radical scavenging activity. Thus, sodium alginate-coated liposomes serve as a promising delivery system for encapsulating DCPH both during storage stability and in the gastrointestinal digestion system.

## 1. Introduction

Recent years have witnessed a surge in the functional food market, underpinned by a shift in consumer focus towards dietary-mediated health [1]. Consequently, bioactive components are no longer viewed as mere supplements but as pivotal factors that positively influence human health [1]. Peptides derived from alternative proteins, especially edible insects, have emerged as potent candidates for health-promoting supplements [2,3]. They exhibit a multifaceted range of biological activities, including immunomodulatory and anticancer effects, etc. [4,5]. The PHGAP and VGPPQ peptides obtained from crickets (*Gryllodes sigillatus*) could be the angiotensin-converting enzyme inhibitor by binding to the enzyme active site residues [6]. Summart et al. [5] reported that the peptides (Pro-Thr-Phe-Leu-Gly-Met-Phe-Leu-Tyr-Glu-Tyr-Ala-Arg) identified from cricket (*Acheta domesticus*) protein hydrolysate exhibited anticancer activity. Also, tetrapeptides derived from cricket protein hydrolysate with Phe-Tyr-Asp-Gln and Phe-Val-Glu-Gly sequences possessed a protective effect against oxidative damage induced by the UVB irradiation of human keratinocyte cells [7]. Additionally, a previous in vitro study indicated that cricket hydrolysate had an excellent safety profile, exhibiting negligible cytotoxicity towards Caco-2 intestinal cells [8]. Apart from these bioactivities, the safety concern regarding the initial materials is necessary for consideration before further processing or application, especially microbial contamination. Generally, postharvest processes, such as boiling or drying, can reduce the microbial load of raw materials [9,10]. Moreover, some processing methods, such as the extraction of antimicrobial peptides from edible insects, could be a promising approach to reduce the load of microorganisms [11,12]. In particular, cysteine-rich peptides (coprisins) from insect protein hydrolysate have been shown to upregulate coprisin genes after bacterial infection, resulting in the inhibition of *S. aureus* and *E. coli* [11]. Thus, the biological safety risk of edible insects could be reduced by producing protein hydrolysates with antimicrobial activity. Even though insect-derived peptides possess various bioactivities, their stability remains a critical challenge. In general, crickets have a high amount of lipids (10–23%) [13], which are highly susceptible to lipid oxidation. As a result, the change in product quality leads to off-odors and discoloration during storage. Thus, the defatting of cricket powder using ethanol with the aid of vacuum impregnation (VI) was conducted via hydrodynamic and relaxation and compression phenomena before preparing protein hydrolysates [8]. The VI process, a non-thermal processing, has been widely used for various pretreatment steps in food processing due to its short processing time and its ability to protect heat-labile compounds from degradation during extraction [14,15] compared to the Soxhlet extraction method [16]. In addition, it is a simple technique that is easy to operate [17], which results in a high yield of extraction that can be obtained at a lower cost than supercritical fluid extraction [18]. Therefore, the VI process was selected as an appropriate method for the defatting of cricket power in this study, which could remove approximately 87% of the lipids [8]. Lowering the lipid content of the initial material possibly retards the change in oxidation of the cricket protein [19]. As a consequence, product quality, both sensory properties and the bioactivity of cricket-derived peptides, could be maintained. Enzymatic hydrolysis, especially using Alcalase and Flavourzyme, was commonly adopted to prepare high-bioactivity cricket protein hydrolysates. Recently, Chotphruethipong et al. [8] prepared a cricket protein hydrolysate using Alcalase hydrolysis. The obtained peptides had a higher α-amino group content (6.22–6.27 mmol L-leucine/g sample) compared to those prepared using Flavourzyme hydrolysis (4.73–4.85 mmol L-leucine/g sample). Thus, Alcalase was deemed an appropriate enzyme for preparing the defatted cricket protein hydrolysate (DCPH). Although cricket-derived peptides have various advantages, their stability and absorption efficiency are still restricted due to the loss of bioactive compounds during storage and gastrointestinal (GI) digestion [20,21]. To overcome this drawback, encapsulation is a potent approach to maintain the functions of DCPH bioactive peptides. Previously, electrospraying and spray-drying techniques have been used to encapsulate *Tenebrio molitor* protein hydrolysate, with pullulan used as encapsulating material [22].

Liposomal vesicles represent an ideal encapsulation matrix for peptides, owing to their amphiphilic architecture, which affords exceptional biocompatibility and a high loading capacity [23]. Moreover, it has been known to be a non-toxic material for delivery systems [23]. Using liposomes to load edible insect-derived peptides could improve bioactivity. Also, liposomal encapsulation could be an effective method for reducing the bitterness of protein hydrolysate [24]. Although liposomes have many advantages, they are prone to oxidation during storage [25]. Also, instability may occur, which frequently leads to vesicular leakage [23]. The aforementioned problems can be solved by coating the liposome with a suitable material. Several coating agents have been used for the enhancement of the stability of liposomes, including polysaccharides and proteins due to their biocompatibility, lack of toxicity, and bio-renewability [26,27]. Many studies used chitosan to coat liposomes, since it could protect against the degradation of bioactive compounds and serve as a non-toxic polymer [28,29,30]. Apart from chitosan, gellan gum (GG), an anionic polysaccharide, has also been used for liposome coating. It is utilized as a thickening agent and emulsifier in both food and pharmaceutical industries [30]. Sodium alginate (SA) is another promising compound to increase the stability of liposomes loaded with peptides both during in vitro digestion and the storage period. SA is able to dissolve in water and provides high bio-compactivity to intestinal cells [31], which more likely increases absorption. Among these compounds, SA- and chitosan-coated liposomes showed higher stability than the GG-coated liposome [30]. Nevertheless, chitosan has some limitations such as low solubility at a neutral pH, swelling, poor thermal stability, and potential reduction in drug entrapment efficiency owing to structural changes [29]. Therefore, the use of SA for coating liposomes may prevent the loss in potency of DCPH bioactive peptides. However, the levels of coating agents used plausibly possess varying efficiency in stabilizing liposomes or the core loading capacity. Also, there is no information on the stability and bioactivities of liposomes containing different levels of DCPH and the effect of coating the liposome loaded with insect peptides with various levels of SA. Therefore, this study aimed to evaluate the effects of encapsulated DCPH and the sodium alginate (SA) coating at different levels on encapsulation efficiency, stability during storage, and the bioavailability of DCPH peptides via Caco-2 cells after gastrointestinal digestion.

## 2. Materials and Methods

### 2.1. Materials and Enzyme

Alcalase (Lot no. 6EA2092) was obtained from Siam Victory Chemicals Co., Ltd. (Bangkok, Thailand). Soy lecithin, cholesterol, and the chemicals used for antioxidant assays, including 2,2-diphenyl-1-picrylhydrazyl (DPPH), 2,2′-azino-bis (3-ethylbenzthiazoline-6-sulphonic acid) (ABTS), and Trolox, were supplied by Sigma-Aldrich (St. Louis, MO, USA). Sodium alginate was given by Sigma-Aldrich. Human colon adenocarcinoma cells (Caco-2 cells, HTB-37^TM^, ATCC, Manassas, VA, USA) with the passage numbers 22–23 were used. Fetal bovine serum (FBS) and Eagle’s minimum essential medium (EMEM) were obtained from Gibco^®^, Thermo Fisher Scientific, Inc. (Waltham, MA, USA).

### 2.2. Preparation of Cricket Powder

Frozen crickets were sourced from a farm in Ubon Ratchathani, Thailand. The samples underwent rigorous cleaning and were subsequently dehydrated using a tray dryer at 50 °C for 15 h [8,32]. The dried material was then ground and sieved through a 60-mesh screen to obtain a fine powder [8]. The resulting powder was then kept at −20 °C for a maximum period of 1 month.

### 2.3. Defatting of Cricket Powder

To remove lipids, the cricket powder was subjected to vacuum impregnation with 95% ethanol for four cycles (cumulative time of 2 h). The defatted powder was then recovered from the solvent and dried until it was entirely free of residual ethanol [8].

### 2.4. Determination of Enzyme Activity

Alcalase activity was evaluated as detailed by Chotphruethipong et al. [8]. Conditions were tested at pH 8.0 and 50 °C for 15 min, for which casein was used as the substrate. One unit of activity was defined as the amount of Alcalase that liberated 0.01 μmol of tyrosine per min (μmol Try/min).

### 2.5. Preparation of Defatted Cricket Protein Hydrolysate (DCPH)

The defatted powder was prepared as a protein hydrolysate through Alcalase hydrolysis. The process was conducted under the following optimal conditions: enzyme concentration of 0.2 units/g dry sample, a temperature of 50 °C with pH 8 for 120 min of hydrolysis [8]. After hydrolysis, the resulting hydrolysate was centrifuged to remove indigestible materials [8]. The supernatant was lyophilized [8]. The resulting protein hydrolysate was used as core bioactive material for the subsequent liposomal preparation. It exhibited the α-amino group content of 6.27 mmol L-leucine/g sample, a size distribution of 493–7248 Da, and a yield of 20.01%, respectively [8].

### 2.6. Preparation of DCPH-Encapsulated Liposome

Liposome was fabricated using the film hydration method. Firstly, soy lecithin and cholesterol were dissolved in absolute ethanol (20 mL) at a 4:1 molar ratio (60 µmol/mL) [23]. The mixture was heated in a water bath (Memmert, W350, Schwabach, Germany) at 50 ± 2 °C. Subsequently, the solvent was removed at 50 ± 2 °C using a rotary evaporator (RC909, Scientific Promotion Co., Ltd., Bangkok, Thailand) until a thin film formed and was dried in a desiccator overnight. The dried films were dispersed in 20 mL of distilled water containing DCPH (1–4% *w*/*v*). The mixture was subjected to sonication in an ultrasonic bath (Taisite, Tianjin, China, UC-G2, 240 W, 40 KHz) for 15 min. The formed liposomes were subsequently evaluated for their encapsulation efficiency (EE).

The unencapsulated DCPH was fractionated from the DCPH-loaded liposomes through the addition of cold acetone, followed by centrifugation at 5000× *g* for 30 min [33]. The resulting supernatant was pooled, and the solvent evaporated in an oven at 60 °C. The unencapsulated fractions were reconstituted in 2 mL of distilled water. To quantify the encapsulated fraction, 0.5 mL of the liposomal suspension was lysed using 0.06% Triton X-100 (1 mL). The protein concentration was subsequently determined via Bradford’s assay [34]. The EE was calculated using Equation (1):

(1) $$EE\;(\%)=\left[\frac{Total\;protein\;content-The\;amount\;of\;free\;protein}{Total\;protein\;content}\right]\times 100$$

The sample having the highest EE was selected for coating with sodium alginate and stability studies.

### 2.7. Preparation of Sodium Alginate-Coated Liposomes

The selected liposome was mixed with sodium alginate (SA) solution at various levels (0.1–0.5%, *w*/*v*) at a ratio of 1:1 (*v*/*v*) under magnetic stirring for 20 min at 28 ± 2 °C, in which the range of SA concentrations used was obtained from a preliminary study. The mixture was then delivered dropwise via a peristaltic pump (Watson marlow digital 505s, Scientific Promotion Co., Ltd., Bangkok, Thailand) and left at 28 °C for 1 h before characterization [31].

#### 2.7.1. Characterization

##### EE, Particle Size (PS), Polydispersity Index (PDI), and Zeta (ζ) Potential

The EE of all samples was evaluated in accordance with the aforementioned methodology. For the characterization of PS, PDI, and Zeta potential, samples were subjected to a 20-fold dilution with distilled water. The resulting suspensions were maintained between pH 6.7 and 6.8 before analysis via a ZetaPlus analyzer (Brookhaven, Holtsville, NY, USA) [35]. Measurement was performed at 25 °C and a scattering angle of 90°. PS, PDI, and Zeta potential were determined in triplicate. To provide a robust comparative framework, uncoated liposome was prepared as a control. SA-coated liposome with the highest EE and the control were lyophilized, which were used for further characterization and storage stability.

##### Morphology of the Selected Liposomes

The selected liposomes were visualized for their morphology using transmission electron microscopy (TEM) (Talos F200i, Thermo Fisher Scientific, Brno, Czech Republic) [35].

The morphological characteristics were further visualized using Confocal Laser Scanning Microscope (CLSM) (LSM 800, Carl Zeiss Microscopy GmbH, Jena, Germany). Both the SA-coated liposomes and the control group were incubated with a 1:1 (*v*/*v*) mixture of 1% Nile blue A and 1% fluorescein-5-isothiocyanate (FITC) for a duration of 10 min. The CLSM was operated at an excitation wavelength of 488 nm and at an emission wavelength of 543 nm. Subsequently, the resulting green and red fluorescent micrographs were captured as images [35].

##### Fourier Transform Infrared Spectroscopy (FTIR) Spectra

The FTIR spectra of lyophilized liposomes, both the selected and control samples, were detected using an FTIR spectrometer (Bruker Equinox 55, Bruker Co., Ettlingen, Germany). Spectral analysis was conducted across a wavenumber range of 400 to 4000 cm^−1^, enabling the identification of functional group vibrations and structural interactions [36].

### 2.8. Storage Stability of the Selected Liposomes

The designated liposomes were transferred to 15 mL conical tubes and kept at 4 °C for a 24-day storage period. At designated intervals (0, 6, 12, 18, and 24 days), the samples were taken for determining EE, while physical parameters including PS, PDI, and Zeta potential were assessed at each interval.

### 2.9. Bioavailability of the Digests Derived from Gastrointestinal Tract (GIT) Across Caco-2 Monolayer

The lyophilized liposomes were subjected to simulated GIT digestion according to the protocol established by Ketnawa et al. [37]. The obtained digests were filtered through a 0.22 µm sterile filter. Before testing bioavailability, Caco-2 cells (1 × 10^4^ cells/well) were treated with the digests at several concentrations (10–250 µg/mL). Cells were subsequently incubated for 24 h [35], and cytotoxicity was then evaluated via MTT assay [38]. Cell viability was reported as % of control. For the transport study, Caco-2 cells (5 × 10^4^ cells/cm^2^) were seeded onto transwell inserts (12 well, 0.4 µm pore size). The culture medium was replenished every 2–3 days over a 24-day differentiation period. Monolayer integrity was rigorously monitored using a Transepithelial Electrical Resistance (TEER) Voltohmmeter (Millicell-ERS, Millipore, County Cork, Ireland), where monolayers with TEER values over 250 Ω·cm^2^ were deemed suitable for transport study.

For bioavailability assessment, 1.5 mL of serum-free media and 50 µL of digests at the optimal concentration were added to the apical side of the transwell plate. Serum-free media (2 mL) were added to the basolateral side. Following a 2 h incubation period, integrity of the monolayer was verified via TEER measurement. The medium from the basolateral part was collected for quantification of protein content (P) and antioxidant activities (A) (DPPH and ABTS radical scavenging activities) [39] to assess peptide transport. Bioavailability was calculated according to Equation (2):

(2) $$Bioavailability\;(\%)=\frac{(P/A)basolateral}{(P/A)added\;digest}\times 100$$

### 2.10. Statistical Analysis

Data derived from triplicate trials were reported as average ± standard deviation (SD). The results were subjected to analysis of variance (ANOVA), and statistically significant differences between means were analyzed using Duncan’s multiple range test [40]. All statistical inferences were made at a significance level of *p* < 0.05.

## 3. Results

### 3.1. Impact of Defatted Cricket Protein Hydrolysate (DCPH) at Different Levels on Encapsulation Efficiency (EE) of Liposomes

The encapsulation efficiency (EE) of liposomes loaded with DCPH fluctuated between 74.55% and 88.18% among the various concentrations tested (Table 1). Different amounts of DCPH significantly affected the EE of liposomes (*p* < 0.05), and the highest EE was observed for liposomes loaded with 2% (*w*/*v*) DCPH. Increased amounts of DCPH (>2%) led to decreases in the EE values of liposomes (*p* < 0.05). Thus, the level of encapsulated DCPH is a crucial parameter in determining the EE of liposomes.

### 3.2. Effect of Sodium Alginate Solution at Different Levels on Encapsulation Efficiency (EE), Particle Size, Polydispersity Index (PDI), and Zeta Potential of Liposomes

The EE values for all liposomal formulations are detailed in Table 2. Uncoated liposomes (L-2%DCPH) exhibited EE levels comparable to those of the sodium alginate (SA)-coated variants, except with SA levels at 0.1% and 0.4% (*p* < 0.05). Notably, the 0.3% (*w*/*v*) SA coating yielded the highest EE (88.39%) compared to other samples (*p* < 0.05). This enhancement in EE is likely attributed to the improved rigidity of the liposomal wall, which increased the loading capacity of DCPH in liposomes. Regarding the particle size (PS) of the vesicles (Table 2), SA-coated liposomes (SA-L-2%DCPH) exhibited a significantly greater PS compared to the uncoated liposome (L-2%DCPH) (*p* < 0.05). PS increased positively with increasing SA concentrations (*p* < 0.05), indicating a concentration-dependent thickening of the polymer layer. No significant differences in PDI were observed among SA-coated liposomes at concentrations ranging from 0.1 to 0.3% (0.62–0.7) (*p* > 0.05) (Table 2), suggesting that the initial coating process maintained a consistent degree of particle dispersion. Interestingly, a lower PDI value was found with increasing SA levels at 0.4–0.5% (0.48–0.51). For the Zeta potential (Table 2), all of the liposomes had a negative surface charge (NSC) (>−30 mV), which indicates that the instability of liposomes occurred to some degree. The SA-coated liposomes demonstrated a significantly higher NSC compared to the uncoated liposomes.

### 3.3. TEM and CLSM Images of the Selected Liposomes

**Figure 1:**
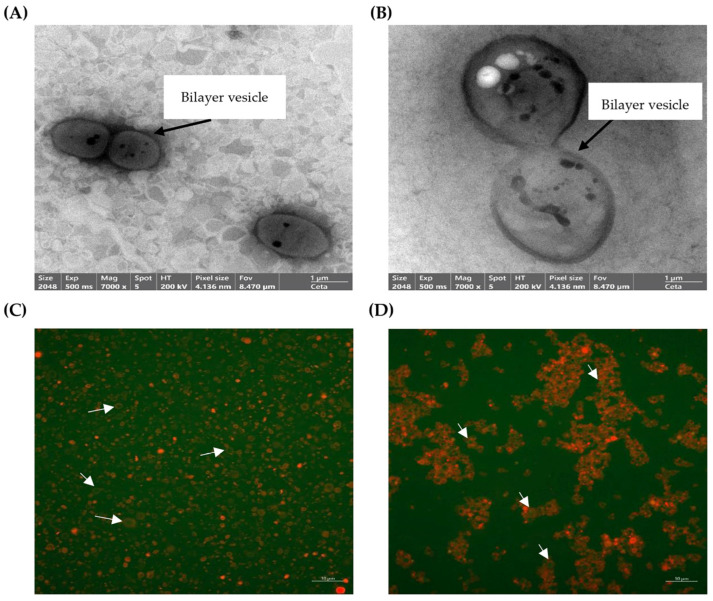
TEM images of liposome loaded with DCPH at 2% (*w*/*v*) (L-2%DCPH) (**A**) and DCPH-loaded liposome coated with sodium alginate at 0.3% (*w*/*v*) (SA-L-2%DCPH) (**B**), and CLSM images of L-2%DCPH (**C**) and SA-L-2%DCPH (**D**). FITC is used to stain peptides present in DCPH, which are detected in green. Nile blue A is used for staining the lipid wall of liposomes, indicated by the red color. White arrows: DCPH loaded with liposomes.

TEM and CLSM images of liposomes without (L-2%DCPH) and with the subsequent coating using SA (SA-L-2%DCPH) are illustrated in Figure 1. Both samples had a spherical morphology (Figure 1A,B). The size of the SA-L-2%DCPH was apparently larger than the L-2%DCPH counterpart, which correlates with the particle size data (Table 2), indicating that SA coating increases liposome size. Moreover, the internal core of the liposomes encapsulated with DCPH was detected (Figure 1A,B), which was confirmed from the CLSM images (Figure 1C,D). The SA-L-2%DCPH had a higher wall thickness than the L-2%DCPH, while the DCPH peptides (green color) were encapsulated in the liposome (red color).

### 3.4. Fourier Transform Infrared Spectroscopy (FTIR) Spectra

**Figure 2:**
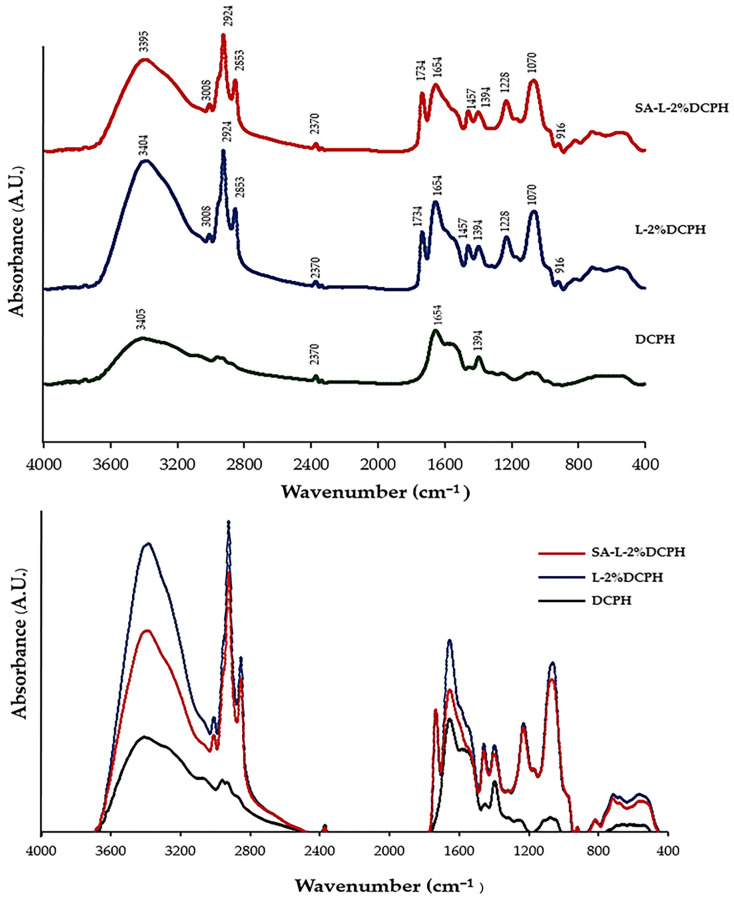
FTIR spectra of DCPH, liposome loaded with DCPH (L-2%DCPH), and DCPH-loaded liposome coated with sodium alginate at 0.3% (*w*/*v*) (SA-L-2%DCPH).

FTIR spectra of the selected liposomes (L-2%DCPH and SA-L-2%DCPH) and DCPH are presented in Figure 2. The amide peaks at ~1654 cm^−1^ (amide I, illustrating C=O stretching vibration) [41] and ~1394 cm^−1^ (amide III, representing C-H bending vibrations) [42] appeared for all of the samples. When DCPH was loaded into a liposome, new peaks appeared at ~3008 cm^−1^ (C–H stretching of the cis-double bond) [43], ~2924 cm^−1^, and ~2854 cm^−1^ (C–H band vibrations of the CH_2_ group) [44]. Also, the absorption peaks appeared at ~1734 cm^−1^ (C=O stretching vibration) [45] and ~1228 cm^−1^ (C=O stretching vibration of phospholipid) [46]. The absorption peaks at ~1457 cm^−1^ and ~1070 cm^−1^ were also detected in the encapsulated samples, corresponding to the CH_3_ scissoring vibration [47] and the stretching of PO_2_ [45]. Moreover, the peak of the O-H stretching vibration was found at a lower wavenumber for DCPH-loaded liposomes (3395 cm^−1^) [48] as compared to that of DCPH (3405 cm^−1^). With coating by sodium alginate (SA), the spectra showed similar characteristic absorption peaks to those of the liposome without coating by SA (L-2%DCPH). Furthermore, a decrease in the intensity of amides A (3200–3600 cm^−1^) and the peaks at wavenumbers below 3200 cm^−1^ of the SA-L-2%DCPH were found to be more pronounced when compared to L-2%DCPH (Figure 2).

### 3.5. Effect of Storage Times on Stability of the Selected Liposomes

#### 3.5.1. EE

EE values of the L-2%DCPH and the SA-L-2%DCPH samples during storage at 4 °C for 24 days are shown in Table 3. At the beginning of the storage duration, both samples had a similar EE. Decreasing EE was observed with the increasing storage period (*p* < 0.05). Higher EE values were found for the SA-L-2%DCPH compared to the L-2%DCPH counterparts throughout the storage time (*p* < 0.05). This finding showed the efficacy of sodium alginate (SA) as a protective barrier, effectively mitigating the leakage of the DCPH peptides from the liposomal core. No difference in EE was found for the SA-L-2%DCPH during the first 6 days of storage (*p* > 0.05). Subsequently, EEs decreased until the end of storage (*p* < 0.05). Thus, the incorporation of a polymeric coating agent serves as a determinative variable in bolstering the shelf life and structural stability of liposomal delivery systems, particularly during prolonged storage.

#### 3.5.2. Particle Size (PS)

PSs of the L-2%DCPH and the SA-L-2%DCPH samples during storage at 4 °C for 24 days are presented in Table 3. The PSs of both samples increased with increasing storage time (*p* < 0.05). Nevertheless, there was no change in the PS of both samples after day 6 of storage (*p* > 0.05). The liposome without SA coating had a smaller PS than that with SA coating at all of the storage times (*p* < 0.05). The results are related to the decreased EE values of both samples when the storage time was increased (Table 3). Thus, storage time had an impact on the particle size of the liposomes.

#### 3.5.3. Polydispersity Index (PDI)

As shown in Table 3, the PDI of both uncoated (L-2%DCPH) and sodium alginate-coated (SA-L-2%DCPH) liposomes has a similar trend throughout the storage period. Nevertheless, both samples exhibited a significant reduction in PDI values relative to their initial day 0 (*p* < 0.05). Also, no change in PDI was found for both samples throughout the storage period at 4 °C. These findings suggest that both the L-2%DCPH and SA-L-2%DCPH liposomes maintained a high degree of colloidal homogeneity and a stable size distribution to some extent under the tested storage conditions.

#### 3.5.4. Zeta Potential

The Zeta potential of the selected liposomes including L-2%DCPH and SA-L-2%DCPH is shown in Table 3. Commonly, the Zeta potential is an indicator of physical stability within the liposome. After preparing liposomes (day 0), both samples had a negative surface charge (NSC). A higher NSC was found in the SA-L-2%DCPH compared to that of the L-2%DCPH (*p* < 0.05), resulting from coating agents, which were localized around the liposome. When the storage time was prolonged, the NSC of both samples decreased (*p* < 0.05) due to loss in the electrostatic repulsion of liposomes during the increased storage time. Higher NSC was found for the SA-L-2%DCPH than for that of the L-2%DCPH, indicating a stronger electrostatic repulsive interaction between liposomes, which prevents them from aggregation. At the end of storage, the NSC of both samples slightly increased (*p* < 0.05), suggesting the instability of liposomes with extended storage times.

### 3.6. Cell Viability and Bioavailability of the Digests of the Selected Liposomes Derived from the GIT Across Caco-2 Monolayer

#### 3.6.1. Cell Viability

**Figure 3:**
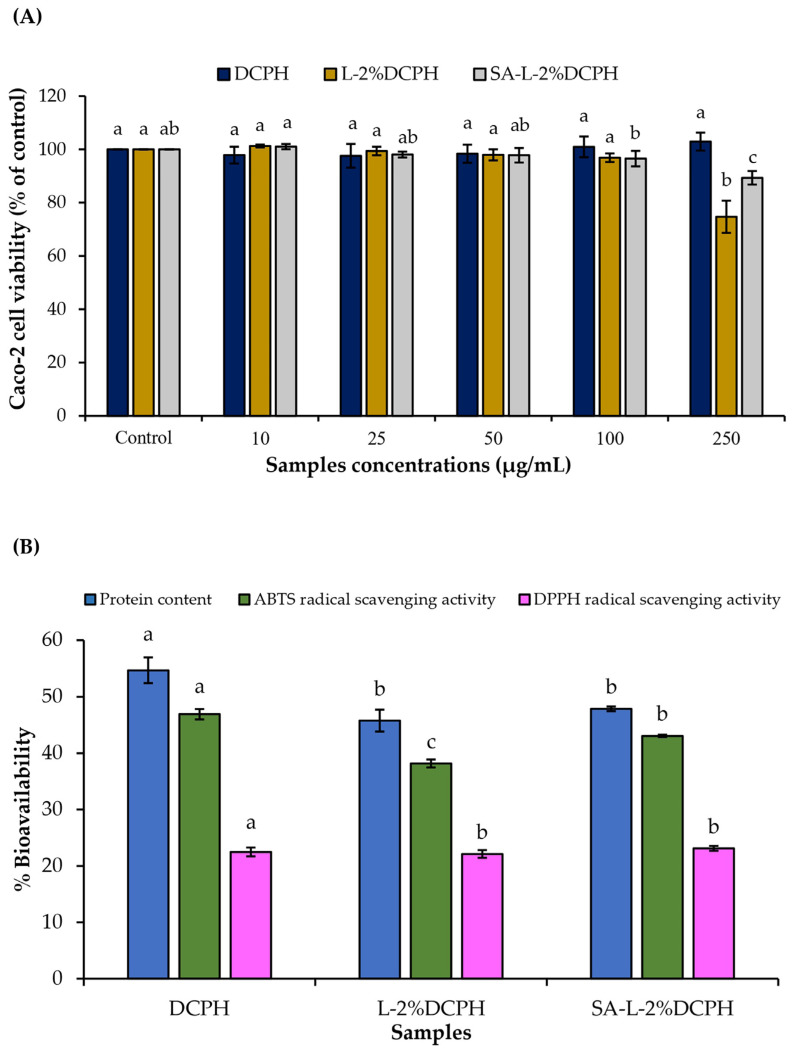
Caco-2 cell viability (**A**) and bioavailability (**B**) of the digests obtained from GIT system. Bars represent the standard deviation (*n* = 3). Different letters on bars indicate significant differences among concentrations or samples within the same assay tested (*p* < 0.05). Control: cells without any treatments.

The effect of the digest at different concentrations on the viability of Caco-2 cells is presented in Figure 3A. All tested concentrations of the digest had no cytotoxicity compared to the control (*p* > 0.05), except at 250 μg/mL. The result showed that the digests had high biocompatibility with epithelial Caco-2 cells. Treatment with the digests at high doses (>100 μg/mL) tended to decrease cell viability. Thus, the maximum concentration of all the digests selected for estimating bioavailability was at 100 μg/mL.

#### 3.6.2. Bioavailability Study

Bioavailability of the digests obtained from GIT is shown in Figure 3B. Protein content and antioxidant activities (ABTS and DPPH radical scavenging activities) of both liposomes were decreased compared to those of DCPH. This indicated that some peptides with antioxidative activities were absorbed into the cells to some extent. Compared to the L-2%DCPH, the SA-L-2%DCPH showed higher bioavailability, as determined by the ABTS radical scavenging activity assay (ABTS-RSA) (*p* < 0.05). However, similar bioavailability was found between both samples when the DPPH radical scavenging assay (DPPH-RSA) was used (*p* > 0.05). It was noted that DCPH peptides with hydrophilic amino acids (AAs) could permeate via Caco-2 cells more efficiently than those with hydrophobic AAs, as shown by the higher ABTS-RSA. Also, the liposome with SA coating could carry hydrophilic peptides into cells effectively compared to the uncoated liposome (*p* < 0.05). Thus, the increased bioavailability of antioxidative peptides was governed by the use of SA as the coating agent on the liposome.

## 4. Discussion

This study prepared liposomes using soy lecithin and cholesterol as the lipid wall by the thin-film method. After loading DCPH-obtained peptides at various levels (1–4% *w*/*v*) (Table 1), liposomes loaded with 2% (*w*/*v*) DCPH showed the highest EE. Increasing levels of DCPH higher than 2% decreased the EE values of liposomes (*p* < 0.05), which might be due to the reduced electrostatic repulsion between phosphate groups of the liposomes and unencapsulated peptides. This reduction in repulsive forces may have caused partial charge neutralization, resulting in liposome aggregation [23]. With excessive amounts of peptides, the vesicles could not entrap all of the peptides in their cores, resulting in structural instability and the leakage of encapsulated peptides. A similar result was reported by Sharma et al. [49] who found that high levels of the salmon frame protein hydrolysate decreased the EE values of liposomes. Thus, loading DCPH at 2% (*w*/*v*) into liposomes (L-2%DCPH) was an optimum level for encapsulation. The EE value of the L-2%DCPH (88.18%) is similar to those reported for the liposomes loaded with spirulina protein hydrolysate (90%) [50] and salmon frame protein hydrolysate (89.63%) [49]. Thus, the interaction between DCPH and lipid membranes is essential for enhancing delivery efficiency in further food application. These findings highlighted the critical role of the amount of DCPH for designing a liposomal formulation in which the use of 2% DCPH provided the maximized EE.

When the L-2%DCPH was coated with SA at various levels (Table 2), there was a noticeable impact on EE. Coating liposomes with 0.3% (*w*/*v*) SA resulted in the highest EE (88.39%) (*p* < 0.05) compared to liposomes coated with other levels. The SA was probably coated on the liposome surface or partially inserted into phospholipid walls, leading to enhanced strength of the liposomal structure. Similarly, astaxanthin-loaded liposomes with chitosan coating exhibited the increased EE due to the presence of chitosan on the surface or its incorporation into the phospholipid bilayer of the liposomes [51]. Thus, the presence of coating materials could improve the EE of liposomes. The result was confirmed by the enhanced NSC and PS (Table 2), which increased with the augmented SA level. Nevertheless, high levels of SA (>0.3%) affected change in NSC. Using SA at low levels (<0.3%) could prevent flocculation and aggregation due to steric hindrance [52], while a high level of SA (<0.3%) caused the decreased NSC, leading to the decreased EE. The results are similar to the study of Wu et al. [31], who applied a 0.3% SA coating to increase the EE value of liposomes containing collagen peptides from 61.52% (uncoated) to 70.99%, while the increase in SA levels resulted in a slight decrease in the EE value. High levels of SA (>0.3%) might enhance carboxyl group (COOH^−^) levels, during which an interaction between the unencapsulated peptides, especially with positively charged AAs, and the COOH^−^ group of SA might occur, as evidenced by the decreased NSC and the increased PS with increasing levels of SA (Table 2). Subsequently, the decreased stability of the colloidal dispersion, which possibly increased the leakage of the encapsulated peptides in the core, led to disruption of the liposome structure or particle aggregation [52]. Pasarin et al. [53] reported that excessively high amounts of SA likely disrupted the gel network, potentially causing liposome aggregation and the release of encapsulated bioactive compounds. Apart from those aforementioned, the PDI indicates the distribution of liposomes, ranging from uniform (homogeneous) to varied (heterogeneous) particles on a scale from 0 to 1 [54]. The SA-coated liposome (0.48–0.7) showed a higher PDI than the uncoated liposome (0.44), reflecting increased size heterogeneity. A high PDI value, particularly >0.7, more likely tended to undergo aggregation/coalescence [23], indicating the instability of liposomes. Thus, all of the liposomes still had some stability. The finding implied that the variety of physical parameters of liposomes was affected by the level of SA used, and therefore, the use of coating materials could be a promising way to enhance the stability of the delivery system though the amount of coating agent that must be carefully controlled. Based on the results shown in Table 2, the optimal level of SA used for coating the liposome was 0.3% (*w*/*v*) in order to provide a high EE without affecting physical stability.

When the morphology of L-2%DCPH and SA-L-2%DCPH was visualized (Figure 1), the structure of both samples was a double bilayer vesicle, as seen in the TEM images (Figure 1A,B). The size of the SA-L-2%DCPH was obviously larger than the L-2%DCPH counterpart (Figure 1A,B), which is similar to the PS data (Table 2), where the SA-L-2%DCPH showed a larger size than the L-2%DCPH (*p* < 0.05). Generally, vesicles with a double bilayer are more stable and better control the release of active compounds than other types [55]. Moreover, the encapsulated liposome contained spherical particles inside the bilayers. These cores might contain peptides in which some hydrophobic domains could form hydrophobic interactions, especially in the bilayer wall, while some hydrophilic AAs with O-H or N-H groups could form hydrogen bonds with the lipid bilayer of the liposome. Also, some positively charged peptides dispersed in the aqueous core or at the liposomal interface might interact with the negative charge of phosphate groups of the bilayers via ionic interactions. Thus, phosphatidylcholine incorporated with cholesterol could be used as liposomal materials, yielding highly stable liposomes. In addition to TEM images, CLSM images confirmed the coating process whereby the SA-L-2%DCPH showed a greater wall thickness than the L-2%DCPH, as indicated by the red fluorescent color (Figure 1C,D). Also, the DCPH peptides were encapsulated in the liposome vehicles, as evidenced by the presence of the fluorescent green color within the lipid wall of the liposome for both samples (Figure 1C,D).

Based on FTIR spectra (Figure 2), no shift in the amide I peak (~1654 cm^−1^) was found for all of the samples tested. Thus, there was no interaction between the phospholipid wall and DCPH core via the C=O group. After encapsulation, there was the presence of new peaks, including at ~3008 cm^−1^, ~2924 cm^−1^, ~2853 cm^−1^, ~1734 cm^−1^, ~1228 cm^−1^, ~1457 cm^−1^, and ~1070 cm^−1^ in the L-2%DCPH and the SA-L-2%DCPH samples. This phenomenon indicated that the phospholipid wall of the liposome might interact with DCPH via several bonds. DCPH contained a high content of hydrophobic AAs (330.36 residues/1000 residues) [8], which might bind to the liposomal wall by H-bonding/hydrophobic–hydrophobic interaction. Also, the cationic AAs present in DCPH, including Arg, Lys, and His [8], plausibly interacted with the negative charge of the phosphate group and the polar region of the liposome, as shown by the new peaks detected. Moreover, absorption of the stretching vibration O-H group band shifted from ~3405 cm^−1^ to a lower wavenumber (3404–3395 cm^−1^) in the encapsulated peptides, which demonstrated that the interactions between the peptides of DCPH and the phospholipid wall were mostly via H-bonding. According to Shishir et al. [56] and Meng et al. [57], the absorption peaks at 2925 cm^−1^, 2854 cm^−1^, and 1735 cm^−1^ belonged to the phospholipid structure [44]. When considering the SA-coated liposome, the reduction in intensities of the amide A (~3395 cm^−1^), amide I (~1654 cm^−1^), and amide III (~1394 cm^−1^) peaks was found compared to the L-2%DCPH. Therefore, the SA coating might have caused changes in the intensities of DCPH peaks. Lower peak intensities, especially at ~1654 cm^−1^, might be due to the interactions between unencapsulated DCPH peptides and the carboxylate anion of SA via the carbonyl group [58]. Also, the shift in peak to a lower wavenumber at ~3395 cm^−1^ of the SA-L-2%DCPH corresponded to the stretching vibration of hydroxyl groups, indicating intermolecular hydrogen bonding between the polysaccharide structure and liposome surface [59]. Furthermore, several overlapping peaks between 1457 cm^−1^ and 916 cm^−1^ were related to COO groups on SA [60] and the C=O stretching vibration of the α-L-guluronic asymmetric ring of SA [61], respectively. The findings confirmed interactions via several bonds formed by the peptides affected by different liposomal formulations.

When the storage stability of the selected liposomes was estimated (Table 3), decreases in EE were found when the storage time increased (*p* < 0.05), reflecting the instability of the liposomes during storage. Nevertheless, higher EE was observed for the SA-L-2%DCPH compared to the L-2%DCPH at all the storage times (*p* < 0.05), suggesting that coating the liposome with SA provided a more effective protection against peptides. The negative charge of SA might have increased repulsions between the liposomes, which decreased flocculation/coalescence. Lower EE was more likely related to the instability of liposomes as a result of aggregation during storage and the loss of flexibility of the liposomal bilayer [23]. When considering the PS of the liposomes (Table 3), there was a relationship with EE (Table 3), which tended to increase with increasing storage times (*p* < 0.05). It demonstrated the aggregation of liposomes as affected by the decrease in electrostatic interaction between liposomes, leading to the release of the encapsulated cargo, particularly peptides with positively charged AAs (from core/unencapsulated). Those peptides likely neutralized the surface charge of liposomes, which increased aggregation; thus, the PS was increased. Apart from PS, the NSC also confirmed the outcomes obtained from EE and PS (Table 3). Reductions in NSC were detected when the storage time was extended. Nevertheless, the SA-L-2%DCPH sample showed higher NSC than the L-2%DCPH sample (*p* < 0.05), reflecting a strong electrostatic repulsive interaction between liposomes, which prevented aggregation. After day 12 of storage, the NSC of both samples slightly increased (*p* < 0.05). Increased NSC was possibly governed by the liberation of peptides with charged AAs entrapped in liposomes, especially peptides with negatively charged AAs, which were localized surrounding liposomes, leading to the increased NSC of liposomes. For the PDI of the selected liposomes (Table 3), both samples had a lower PDI compared to those obtained at day 0 (*p* < 0.05), exhibiting that the storage times affected a significant reduction in PDI values relative to their initial day 0 (*p* < 0.05). The high temperature used for liposomal preparation (28 ± 2 °C) might increase particle collision rates, leading to the increased PDI value [62,63,64], whereas storage at 4 °C could retard phase transitions of the liposomal membrane [64]. PDI is a crucial metric for quantifying the homogeneity of the size distribution [62]. Increasing PDI at the initial day of storage was not only governed by peptides loaded into liposomes but also the temperature used. Higher temperatures could increase the PDI of liposomes by reducing electrostatic repulsion [65]. The result was associated with the PS (Table 3). With the increasing storage period, decreased PDIs of both samples were found, since the low temperature used during storage resulted in the retardation of the coalescence rate to some extent. However, PDI values of both samples did not exceed 0.7 throughout the storage period, demonstrating that they still had a narrow particle size distribution under the temperatures and time used [62].

When the selected liposome was digested via the GIT system, the toxicity of the resulting digests (L-2%DCPH and SA-L-2%DCPH) at various concentrations on Caco-2 cells was investigated (Figure 3A). Adding the digests obtained from both samples at concentrations ranging from 10 to 100 µg/mL did not alter cell viability compared to the control (without adding the digests). Thus, the maximized level of both samples (100 µg/mL) was selected for assessment of bioavailability via the Caoco-2 monolayer. Bioavailability of the digests through Caco-2 cells was assessed by protein content and antioxidant activities (DPPH-RSA and ABTS-RSA) (Figure 3B). Commonly, the bioavailability of bioactive peptides depends on several distinct cellular pathways such as paracellular and transcellular pathways [35]. The DCPH digest permeated the basolateral side more effectively than those obtained from the liposomes, as indicated by the higher protein content and antioxidative activities of the permeates (Figure 3B). This might be due to the higher content of initial peptides compared to those encapsulated in liposomes. Low-molecular-weight peptides, specifically di- and tripeptides, are predominantly transported via the PepT1 route [66]. This pathway is favorable for peptides containing basic amino acid residues, which likely enhance the molecule’s affinity for the PepT1 binding pocket, thereby augmenting its transport efficiency. Furthermore, peptides with high hydrophobicity can facilitate membrane permeation by engaging with the transporter’s hydrophobic sites of PepT1 [67]. The paracellular pathway, the passage between adjacent cells via tight junctions, serves as a primary conduit for oligopeptides. This route is especially conducive to the translocation of small, negatively charged peptides that cannot easily traverse the lipid bilayer. For structurally complex or high-molecular-weight peptides, they are transported via the transcytosis pathway [68], which shows a marked preference for cationic (positively charged) peptides and large lipophilic (hydrophobic) molecules [68]. A comparative study of the Caco-2 cell permeates revealed that the SA-L-2%DCPH digest exhibited significantly higher ABTS-RSA than its uncoated counterpart (*p* < 0.05), suggesting that the SA coating effectively enhanced the transepithelial absorption of bioactive DCPH peptides. These observations align with the findings of Wu et al. [31], who reported that the use of SA could increase the transport of collagen peptides across the Caco2/HT29 co-culture model. The transport of peptides rich in hydrophilic AAs likely proceeds through both paracellular and transcellular pathways across the Caco-2 cell monolayer. Conversely, no differences in protein content and DPPH-RSA between both digests were found (*p* > 0.05). This was probably due to the equilibrium of the concentration gradient of substances between intracellular and extracellular spaces, resulting in high amounts of peptides with hydrophobic AAs that could not diffuse into the basolateral side. Consequently, the transepithelial permeation of the DCPH-derived digests appears to be predominantly mediated by the PepT1 carrier-mediated route and the transcytosis pathway.

## 5. Conclusions

DCPH was successfully integrated into nanoliposomes. The physicochemical properties of the resulting liposomes, including the EE, particle size, polydispersity index, and Zeta potential, were significantly modulated by both the DCPH loading concentration and the presence of an SA coating. Higher EE was achieved for the DCPH-loaded liposome at 2% (*w*/*v*), in which EE was 88.18%. After coating with an SA solution at 0.3% (*w*/*v*), the DCPH-loaded liposome had higher storage stability than the uncoated liposome, as evidenced by the higher EE throughout storage at 4 °C over a 24-day duration. Additionally, antioxidative peptides entrapped in SA-coated liposomes were more stable after transport via Caco-2 cells. Consequently, the encapsulation of bioactive peptides within SA-coated liposomes could be an effective approach for protecting them from degradation during storage and gastrointestinal digestion.

## Figures and Tables

**Table 1:** Encapsulation efficiency (EE) of liposomes loaded with defatted cricket protein hydrolysate (DCPH) at different levels.

DCPH Levels (% *w*/*v*)	EE (%)
1%	74.55 ± 0.28 ^d^
2%	88.18 ± 1.40 ^a^
3%	76.18 ± 0.16 ^c^
4%	77.88 ± 0.44 ^b^

Values are expressed as mean ± SD (*n* = 3). Different letters within the same column indicate significantly different (*p* < 0.05) mean values.

**Table 2:** Effect of sodium alginate solution at different levels on encapsulation efficiency (EE), particle size (PS), polydispersity index (PDI), and Zeta potential of liposomes.

Sodium Alginate Levels (%*w*/*v*)	EE (%)	PS (nm)	PDI	Zeta Potential (mV)
L-2%DCPH	88.18 ± 1.40 ^ab^	562.63 ± 16.61 ^c^	0.44 ± 0.04 ^c^	−10.99 ± 2.14 ^c^
0.1	86.15 ± 0.87 ^c^	617.27 ± 78.43 ^bc^	0.70 ± 0.10 ^a^	−21.93 ± 2.42 ^b^
0.2	86.78 ± 0.64 ^bc^	633.42 ± 42.54 ^bc^	0.64 ± 0.11 ^a^	−24.11 ± 2.01 ^a^
0.3	88.39 ± 0.53 ^a^	637.68 ± 22.47 ^bc^	0.62 ± 0.11 ^ab^	−22.21 ± 0.49 ^ab^
0.4	86.50 ± 0.12 ^c^	687.92 ± 67.36 ^ab^	0.48 ± 0.11 ^bc^	−22.07 ± 2.92 ^b^
0.5	86.85 ± 0.21 ^bc^	727.58 ± 93.60 ^a^	0.51 ± 0.08 ^c^	−22.18 ± 1.41 ^b^

Values are expressed as mean ± SD (*n* = 3). Different letters within the same column indicate significantly different (*p* < 0.05) mean values. L-2%DCPH: liposome loaded with defatted cricket protein hydrolysate at 2% (*w*/*v*) without sodium alginate coating.

**Table 3:** Encapsulation efficiency (EE), particle size (PS), polydispersity index (PDI), and Zeta potential of the selected liposomes during storage at 4 °C for 24 days.

Day	L-2%DCPH				SA-L-2%DCPH			
	EE (%)	PS (nm)	PDI	Zeta Potential (mV)	EE (%)	PS (nm)	PDI	Zeta Potential (mV)
0	88.18 ± 1.40 ^a^	562.63 ± 16.61 ^c^	0.44 ± 0.04 ^a^	−10.99 ± 2.14 ^a^	88.39 ± 0.53 ^a^	637.68 ± 22.47 ^c^	0.62 ± 0.11 ^a^	−22.21 ± 0.49 ^ab^
6	71.23 ± 0.47 ^b^	796.08 ± 83.13 ^b^	0.37 ± 0.02 ^b^	−9.02 ± 2.56 ^ab^	86.84 ± 1.82 ^a^	823.57 ± 25.32 ^b^	0.31 ± 0.01 ^b^	−18.19 ± 2.70 ^c^
12	64.16 ± 0.69 ^c^	873.57 ± 40.96 ^a^	0.35 ± 0.08 ^b^	−7.87 ± 0.90 ^b^	82.95 ± 1.86 ^b^	965.57 ± 61.73 ^a^	0.34 ± 0.08 ^b^	−18.17 ± 2.17 ^c^
18	61.98 ± 0.84 ^d^	876.50 ± 12.45 ^a^	0.34 ± 0.03 ^b^	−8.84 ± 2.44 ^ab^	80.12 ± 0.51 ^c^	970.97 ± 14.57 ^a^	0.37 ± 0.03 ^b^	−21.54 ± 1.89 ^b^
24	56.78 ± 1.94 ^e^	870.47 ± 25.41 ^a^	0.36 ± 0.02 ^b^	−8.37 ± 2.79 ^ab^	76.37 ± 0.53 ^d^	1000.93 ± 16.52 ^a^	0.38 ± 0.02 ^b^	−23.41 ± 1.80 ^a^

Values are expressed as mean ± SD (*n* = 3). Different letters within the same column indicate significantly different (*p* < 0.05) mean values. L-2%DCPH: liposome loaded with 2% (*w*/*v*) defatted cricket protein hydrolysate (DCPH); SA-L-2%DCPH: DCPH-loaded liposome (2%, *w*/*v*) coated with 0.3% (*w*/*v*) sodium alginate.

## Data Availability

The original contributions presented in the study are included in the article; further inquiries can be directed to the corresponding author.

## Abbreviations

The following abbreviations are used in this manuscript:

AAs	Amino acids
ABTS	ABTS radical scavenging activity
DCPH	Defatted cricket protein hydrolysate
DPPH	DPPH radical scavenging activity
SA	Sodium alginate
L-2%DCPH	Liposome loaded with DCPH at 2% (*w*/*v*)
SA-L-2%DCPH	DCPH-liposome coated with solidum alginate
GIT	Gastrointestinal tract
